# Weight loss reduces basal-like breast cancer through kinome reprogramming

**DOI:** 10.1186/s12935-016-0300-y

**Published:** 2016-04-01

**Authors:** Yuanyuan Qin, Sneha Sundaram, Luma Essaid, Xin Chen, Samantha M. Miller, Feng Yan, David B. Darr, Joseph A. Galanko, Stephanie A. Montgomery, Michael B. Major, Gary L. Johnson, Melissa A. Troester, Liza Makowski

**Affiliations:** CB 7461, Department of Nutrition, Gillings School of Global Public Health, University of North Carolina at Chapel Hill, 2203 McGavran Greenberg Hall, Chapel Hill, NC 27599-7461 USA; Department of Pharmacology, University of North Carolina at Chapel Hill, Chapel Hill, NC USA; Mouse Phase I Unit, University of North Carolina at Chapel Hill, Chapel Hill, NC USA; Department of Medicine, University of North Carolina at Chapel Hill, Chapel Hill, NC USA; Department of Pathology and Laboratory Medicine, University of North Carolina at Chapel Hill, Chapel Hill, NC USA; Department of Cell and Developmental Biology, University of North Carolina at Chapel Hill, Chapel Hill, NC USA; Lineberger Comprehensive Cancer Center, University of North Carolina at Chapel Hill, Chapel Hill, NC USA; Department of Epidemiology, University of North Carolina at Chapel Hill, Chapel Hill, NC USA

**Keywords:** Kinome, MAPK, AMPK obesity, Leptin, High fat diet, Adiposity, Body composition

## Abstract

**Background:**

Obesity is associated with an aggressive subtype of breast cancer called basal-like breast cancer (BBC). BBC has no targeted therapies, making the need for mechanistic insight urgent. Reducing adiposity in adulthood can lower incidence of BBC in humans. Thus, this study investigated whether a dietary intervention to reduce adiposity prior to tumor onset would reverse HFD-induced BBC.

**Methods:**

Adult C3(1)-Tag mice were fed a low or high fat diet (LFD, HFD), and an obese group initially exposed to HFD was then switched to LFD to induce weight loss. A subset of mice was sacrificed prior to average tumor latency to examine unaffected mammary gland. Latency, tumor burden and progression was evaluated for effect of diet exposure. Physiologic, histology and proteomic analysis was undertaken to determine mechanisms regulating obesity and weight loss in BBC risk. Statistical analysis included Kaplan–Meier and log rank analysis to investigate latency. Student’s t tests or ANOVA compared variables.

**Results:**

Mice that lost weight displayed significantly delayed latency compared to mice fed HFD, with latency matching those on LFD. Plasma leptin concentrations significantly increased with adiposity, were reduced to control levels with weight loss, and negatively correlated with tumor latency. HFD increased atypical ductal hyperplasia and ductal carcinoma in situ in mammary gland isolated prior to mean latency—a phenomenon that was lost in mice induced to lose weight. Importantly, kinome analysis revealed that weight loss reversed HFD-upregulated activity of PKC-α, PKD1, PKA, and MEK3 and increased AMPKα activity in unaffected mammary glands isolated prior to tumor latency.

**Conclusions:**

Weight loss prior to tumor onset protected against the effects of HFD on latency and pre-neoplastic lesions including atypical ductal hyperplasia and DCIS. Using innovative kinomics, multiple kinases upstream of MAPK/P38α were demonstrated to be activated by HFD-induced weight gain and reversed with weight loss, providing novel targets in obesity-associated BBC. Thus, the HFD-exposed microenvironment that promoted early tumor onset was reprogrammed by weight loss and the restoration of a lean phenotype. Our work contributes to an understanding of underlying mechanisms associated with tumor and normal mammary changes that occur with weight loss.

**Electronic supplementary material:**

The online version of this article (doi:10.1186/s12935-016-0300-y) contains supplementary material, which is available to authorized users.

## Background

Being overweight or obese contributes to ~20 % of US female cancer deaths, with breast cancer being the most common malignancy and second leading cause of cancer death in females [1–8]. Obese women have a higher risk of invasive breast cancer, developing distant metastasis, recurrence, and mortality [9–14]. Basal-like breast cancers (BBCs) are a subset of triple negative breast cancers (TNBC) that are highly proliferative and metastatic, resulting in poor overall survival [15–17]. Receptor-specific targeted therapies are currently unavailable for TNBC, emphasizing the need for innovative approaches for these highly aggressive subtypes [18]. The role of obesity in BBC is well-established through epidemiologic [17, 19] and experimental findings from our lab and others [20–23], although the underlying mechanisms connecting obesity with BBC occurrence are unknown. The WHO reported that body weight and physical inactivity account for at least 20 % of several of the most common cancers including breast cancer, and posit that more than a third of cancers could be avoided by maintaining a healthy life style. Millikan et al. indicated that up to 68 % of BBC could be prevented by promoting breastfeeding and reducing abdominal adiposity [16]. Therefore, weight loss may have a protective effect on BBC tumorigenesis or tumor progression.

Understanding the reversibility and molecular underpinnings of obesity-induced risk in BBC is necessary to design prevention and treatment strategies. In addition to complex systemic changes associated with obesity, it is possible that alterations to the microenvironment surrounding a pre-cancerous lesion or tumor account for additional obesity-driven regulators of cancer progression [24, 25]. The cellular composition, metabolites, kinases, growth factors including adipokines such as leptin, and modifications to the extracellular matrix in the microenvironment have critical effects on tumor biology [22, 26]. Alterations to the microenvironment may contribute to the conversion of normal epithelial cells to hyperplastic cells and/or drive the progression from atypical hyperplasia to carcinoma [27]. The window of dietary exposures, including weight gain and weight loss, is another important variable to consider in breast cancer risk. Epidemiological studies have shown that weight gain in adult life specifically is associated with increased breast cancer risk [28, 29]. Thus, we hypothesized that early adulthood HFD-induced carcinogenic effects on BBC can be reversed through weight loss and are dependent upon the changes in the microenvironment of mammary glands before tumor onset. To test our hypotheses, we used C3(1)-Tag GEMMs, which have been previously shown to be a faithful preclinical model of human BBC by our lab and others [20, 21, 30, 31]. Herein, we demonstrate that HFD-accelerated latency was delayed by weight loss that was induced by switching from HFD to LFD. Diet regulated changes to the mammary gland microenvironment included significantly increased atypical ductal hyperplasia (ADH) and ductal carcinoma in situ (DCIS) before tumor onset. Weight loss and reduction of adiposity associated with diet switch successfully reversed ADH and DCIS to the levels detected in lean mice. To determine specific pathways regulated by weight loss, unaffected mammary glands isolated prior to average latency were subjected to activated kinome profiling. Relative to HFD-induced kinase changes, weight loss-induced regulation included significantly reduced activity of PKC-α, PKD1, PKA, and MEK3 in unaffected mammary glands, which together result in inactivation of the mitogen-activated protein kinase (MAPK) pathway associated with proliferation. Weight loss after weight gain also increased the activity of metabolically sensitive kinases such as 5′-AMP-activated protein kinase AMPK which can also inhibit proliferative capacity. Our findings suggest that HFD altered the mammary gland prior to frank tumor onset, which contributed to ADH, DCIS and tumor latency, and these changes were limited by weight loss.

## Results

### Tumor latency shortened by HFD exposure could be delayed by weight loss prior to tumor onset

**Fig. 1:**
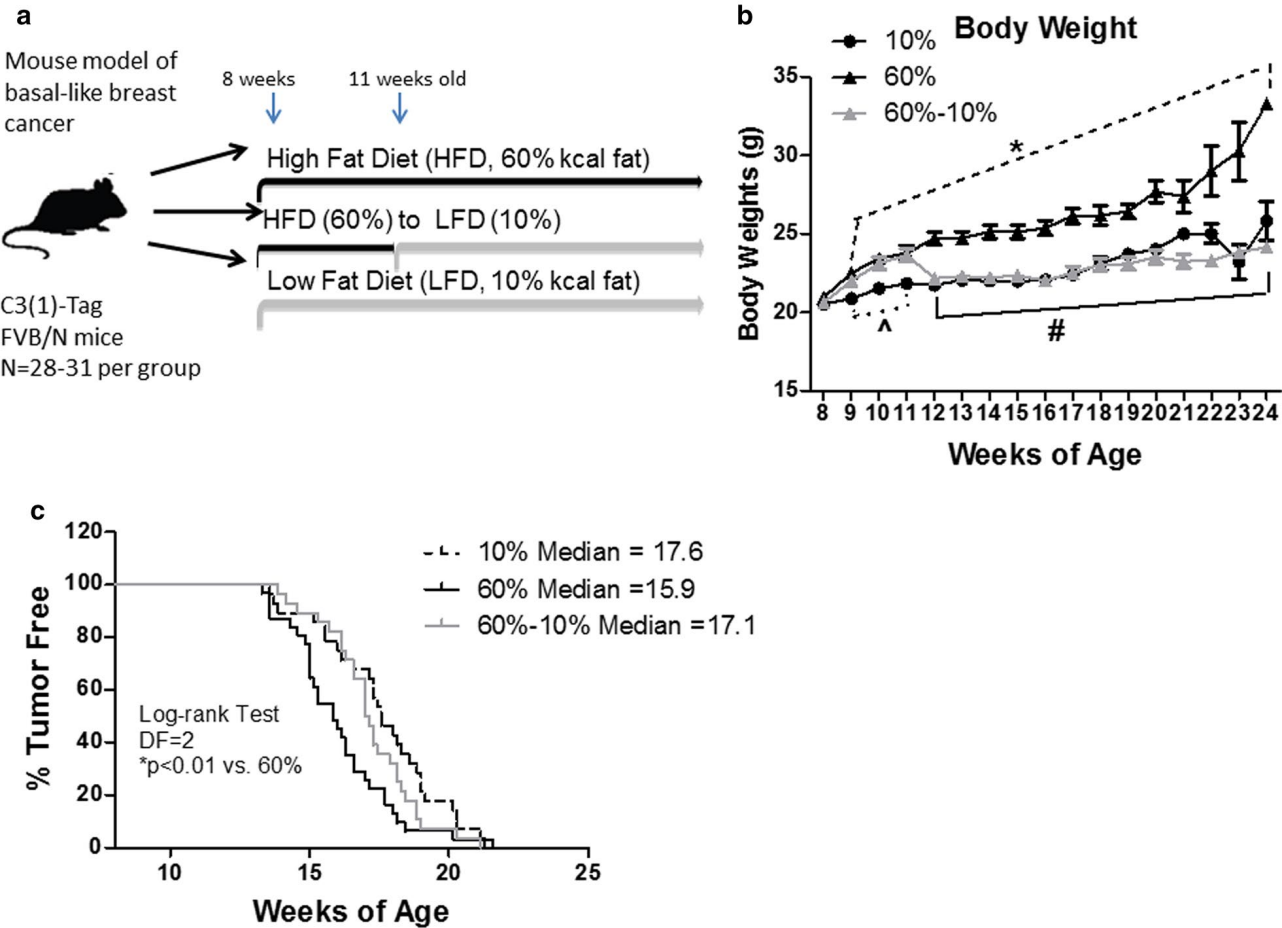
Weight loss protected against HFD-mediated early BBC onset. **a** Model of study design 1. At 8 weeks of age, C3(1)-Tag mice were randomly assigned to 10 % (N = 28) and 60 % (N = 59) diet groups. After 3 weeks on diet, at 11 weeks of age, N = 28 mice on 60 % were switched to 10 % diet. Mice were monitored for tumor onset by palpation 3 times a week. Three weeks after tumor onset, mice were sacrificed. **b** Body weight was measured weekly. 10 % vs. 60–10 % (^^^P < 0.05); 10 % vs. 60 % (*P < 0.01); and 60 % vs. 60–10 % (^#^P < 0.0001). **c** Mice were tumor free until first tumor palpated (P < 0.01, 10 % vs. 60 %; 60–10 % vs. 60 %)

To determine if weight loss in mice fed HFD prior to tumor onset would alter the course of tumorigenesis and/or progression, mice were subjected to various diet exposures (Fig. 1a). At 8 weeks of age, C3(1)-Tag mice were fed control 10 % LFD (N = 28) or 60 % HFD (N = 59). There were no significant differences in body weight before starting the diet study (8 weeks of age, Fig. 1b). Mice on 60 % diet gained more weight than those on control 10 % diet, and were significantly different after 1 week on diet (P < 0.01) and remained significantly different until the end of the study (Fig. 1b). At 11 weeks of age, N = 28 mice on the 60 % diet were switched to 10 % diet to induce weight loss (60–10 % group, Fig. 1a). At week 13 (1 week post diet switch), mice on 60–10 % diet lost weight to the level of 10 %-fed mice, and remained lean, identical to 10 %-fed mice for the remainder of the study. Mice from 60 to 10 % diet group weighed significantly less compared to mice on 60 % diet from week 13 to the end of study (P < 0.0001, Fig. 1b).

Time to first tumor was detected as tumor latency. A log-rank test was performed and Chi square was equal to 6.73, with 2 degrees of freedom. Hazard ratios comparing 60 to 10 % was 0.51 (P < 0.01), 60 to 60–10 % was 1.96, and 10 to 60–10 % was 0.87 (Fig. 1c). Mice exposed to 60 % diet exhibited a significant decrease in median tumor latency compared with lean controls (10 %), from a median of 17.6 weeks in 10 %-fed mice to 15.9 weeks in 60 %-fed mice (P < 0.01, Fig. 1c). Mean latencies were 17.6, 16.1 and 17.2 weeks for mice in 10, 60 and 60–10 % diet groups, respectively (P < 0.05). Mice that first gained weight and then lost weight after diet switch had significantly delayed tumor latency of 17.1 weeks compared to 60 %-fed mice (P < 0.01, Fig. 1c). No significant difference in median or mean tumor latency was found between mice on 10 % diet and on 60–10 % diet. There was no change in total tumor burden (Additional file 1a). Furthermore, no difference was found in the tumor volumes at different time points (Additional file 1a).

**Fig. 2:**
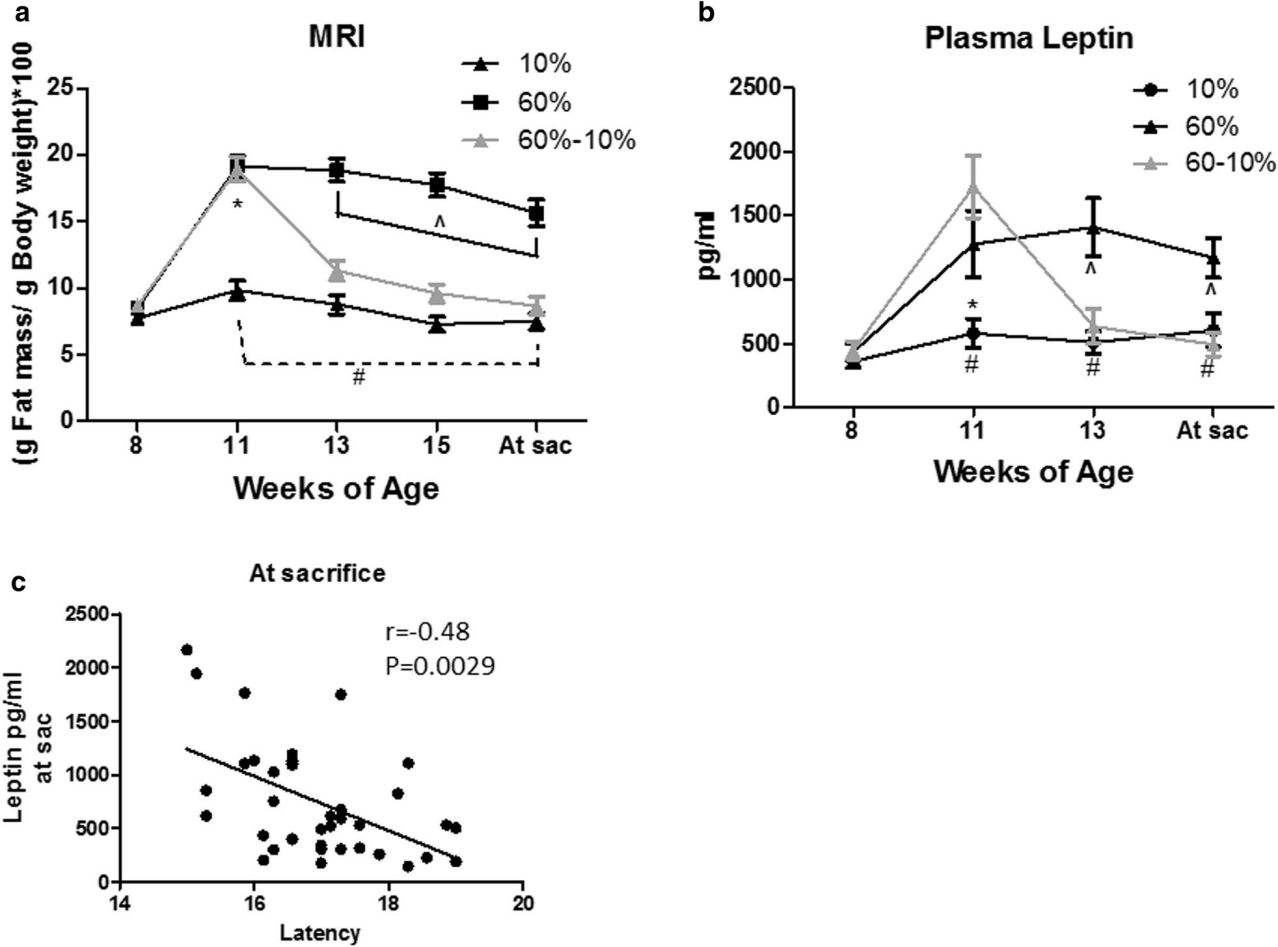
Body composition predicts latency. **a** MRI was used to evaluate body composition. 10 vs. 60 % (^#^P < 0.0001); 60 % vs. 60–10 % (^^^P < 0.0001); and 10 % vs. 60–10 % (*P < 0.0001). N = 28 10 %, N = 31 60 %; N = 28 60–10 %. **b** Plasma leptin was quantified. 10 % vs. 60 % (^#^P < 0.0001); 60 % vs. 60–10 % (^^^P < 0.0001); 10 % vs. 60–10 % (*P < 0.0001). N = 12 10 %, N = 12 60 %; N = 13 60–10 %. **c** Spearman correlations of plasma leptin with latency at sacrifice (P = 0.0029). N = 12 10 %, N = 12 60 %; N = 13 60–10 %

Early adulthood HFD-induced changes in body composition are rapidly reversed by weight loss. Although the mice only gained a few grams on the 60 % HFD, which is typical for FVB/N and C3(1)-Tag mice [20, 21, 32], there was a significant effect of diet exposure and diet switch on body composition. Mice fed the 60 % diet gained body fat in the first 3 weeks (from 8 to 11 weeks of age) as determined by MRI (Fig. 2a). Mice fed 60 % diet had significantly greater body fat mass compared to the mice on 10 % diet at 11 weeks of age (P < 0.0001, Fig. 2a). At 13 weeks of age (2 weeks after diet switch), body fat content of mice on the 60–10 % diet decreased significantly, and remained similar to 10 % fed mice until sacrifice. Mice on 60 % diet exhibited greater body fat content compared to 10 % and 60–10 % fed mice from 13 weeks of age until sacrifice (P < 0.0001 at weeks 13 and 15, and at sacrifice; Fig. 2a). There were no significant differences in absolute lean mass in grams in any of the diet groups tested (data not shown). In line with changes in adiposity, plasma concentrations of the adipokine leptin increased significantly in mice fed the 60 % diet compared to mice fed 10 % diet after 3 weeks on diet (P < 0.0001, Fig. 2b). After diet switch from 60 to 10 % diet, leptin concentrations of mice in the 60–10 % group were significantly reduced to the concentrations in lean mice (P < 0.0001, 60–10 % vs. 60 %; Fig. 2b), consistent with adiposity and body weights. No significant difference in leptin was found between 10 and 60–10 % groups. The 60 %-fed group had significantly greater leptin concentrations than the other 2 groups as soon as 2 weeks after the diet switch and at sacrifice (P < 0.0001, 60–10% vs. 60% and 10 vs. 60 %, Fig. 2b). No correlation of plasma leptin concentration and tumor latency was found before diet start at 8 weeks of age (data not shown). However, at 11 and 13 weeks of age (P = 0.017, 0.0043, respectively, data not shown) and at sacrifice, plasma leptin concentration negatively and significantly correlated with tumor latency (P = 0.0029, Fig. 2c). There were no diet-induced changes in 6-h fasting blood glucose levels (Additional file 2a) or plasma insulin (Additional file 2b) over the course of the study. Likewise, HOMA_IR_, a proxy measure of insulin resistance, did not reveal significant diet effects (Additional file 2c).

**Fig. 3:**
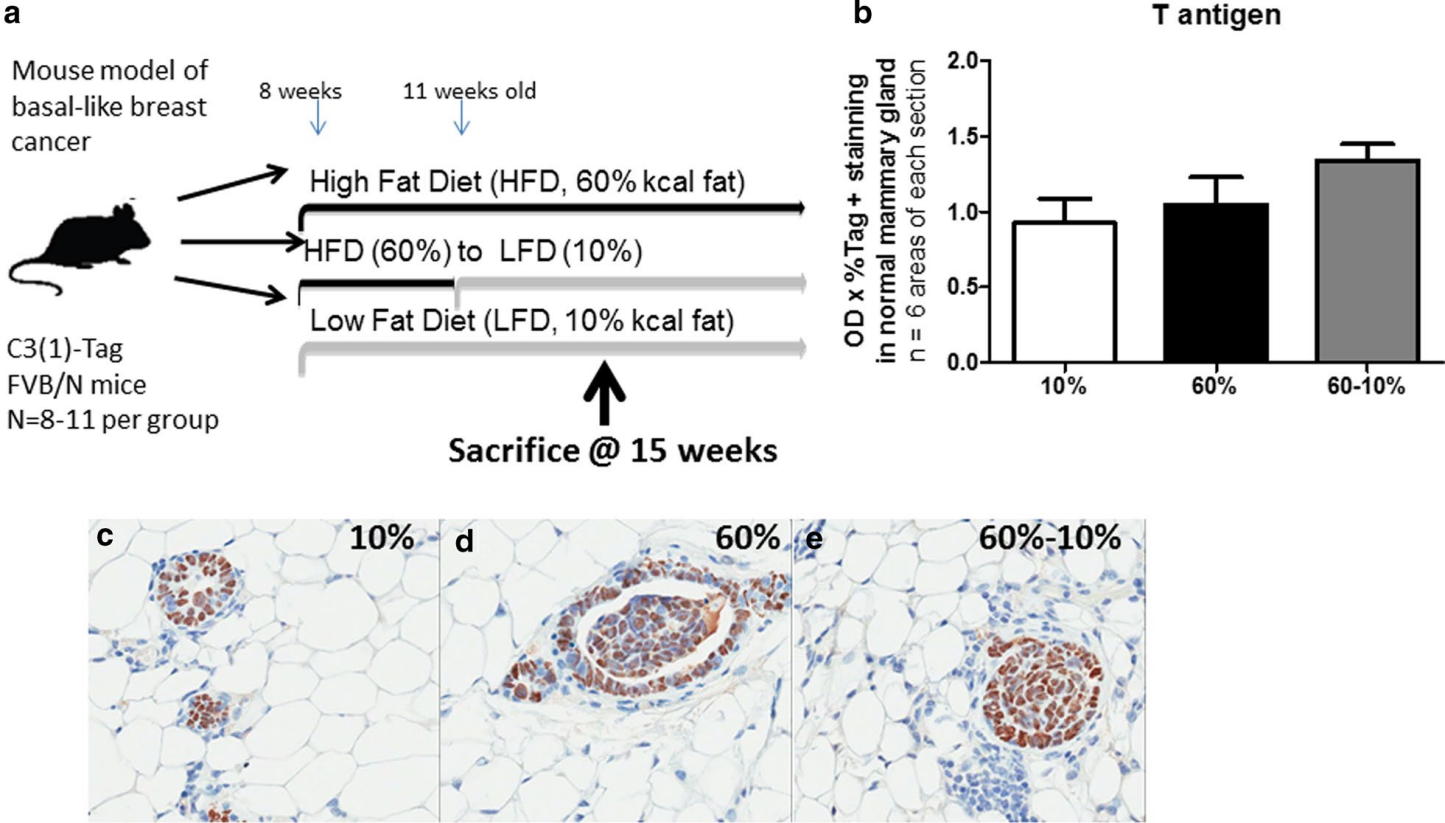
Diet did not affect tumor initiation. **a** Model of study 2. A second set of mice were started on 10 % (N = 8) and 60 % (N = 20) at 8 weeks of age. At 11 weeks of age, N = 11 of 60 %-fed were switched to 10 % as in study design 1. Mice were sacrificed at 15 weeks of age prior to average tumor latency and unaffected mammary glands were isolated. **b** T antigen (Tag) expression was measured in unaffected mammary glands by immunohistochemical staining. **c–e** Representative 40X images are shown of Tag IHC staining from **c** 10 % diet group, **d** 60 % diet group, and **e** 60–10 % diet group

**Fig. 4:**
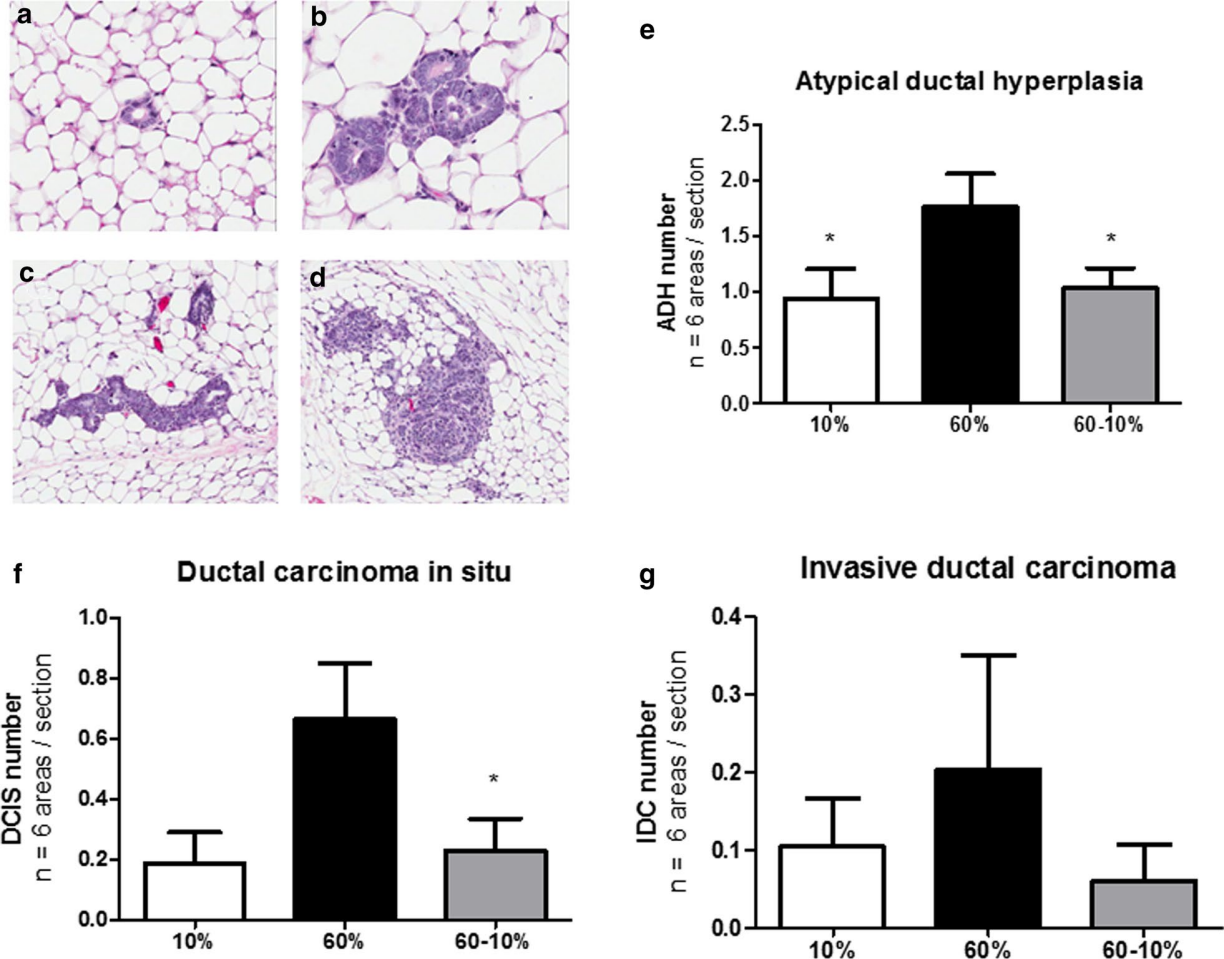
Pathological changes in the unaffected mammary gland induced by HFD were reversed by weight loss. Unaffected mammary glands were collected from 15-week old C3(1)-Tag mice in study 2. **a**–**d**. Representative 40X images are shown of **a** normal duct, **b** ADH, **c** DCIS, and **d** IDC. **e** Areas of ADH were quantified in N = 6, 40X regions per section per mouse. *P < 0.05, 10 % vs. 60 %; 60–10 % vs. 60 %. **f** Areas of DCIS were quantified as in **e** *P < 0.05, 60–10 % vs. 60 %. **g** Areas of IDC were quantified as in **e** N = 8 10 %; N = 9, 60 %; N = 11, 60–10 %

HFD and weight loss affected pre-neoplastic lesions in the microenvironment of unaffected mammary glands. Since HFD exposure and weight loss significantly altered tumor latency in this GEMM that is already transgenically induced, we next sought to examine alterations in the microenvironment of mammary glands prior to palpation of tumor or latency. Mice were placed on diets at 8 weeks of age, and diet switch occurred at 11 weeks of age, as above, however all mice were sacrificed at 15 weeks of age to examine unaffected mammary glands where no tumor was palpated (Fig. 3a). C3(1)-Tag mice develop tumors due to T antigen (Tag) under the control of the rat prostatic steroid binding protein C3(1) gene [33]. It is well-established that at 8 weeks old, C3(1)-Tag mice develop ADH [33]. At 12 weeks of age, DCIS appears, and at 16–18 weeks of age on average, female mice develop tumors when DCIS becomes IDC [33]. Using a well-defined model allowed for us to test for specific alterations to the mammary gland associated with diet exposure. First, we examined if initiation of diet at 8 weeks of age altered the number of cells expressing the SV40 Tag transgene which could regulate latency. Tag immunohistochemical analysis exhibited no significant changes in unaffected mammary glands of mice (quantification in Fig. 3b and representative images in Fig. 3c–e). Secondly, we determined if HFD exposure and diet-induced weight loss altered pre-cancerous lesion formation. Histopathologic analysis of unaffected mammary glands was examined for regions including ADH, DCIS, and IDC (Fig. 4a–d). C3(1)-Tag mice fed the 60 % diet had significantly increased ADH in unaffected mammary glands at 15 weeks of age (P < 0.05, Fig. 4e). Dietary intervention-induced weight loss significantly decreased ADH to the levels detected in mammary glands from lean mice on 10 % diet (P < 0.05, 60 vs. 60–10 %, Fig. 4e). HFD exposure increased DCIS by about threefold; interestingly, weight loss significantly decreased DCIS (P < 0.05, 60 vs. 60–10 %, Fig. 4f). Although mammary glands from mice fed 60 % diet had increased numbers of IDC in unaffected mammary glands compared to 10 and 60–10 %-fed mice, no statistical differences were found between diet exposures (Fig. 4g).

**Fig. 5:**
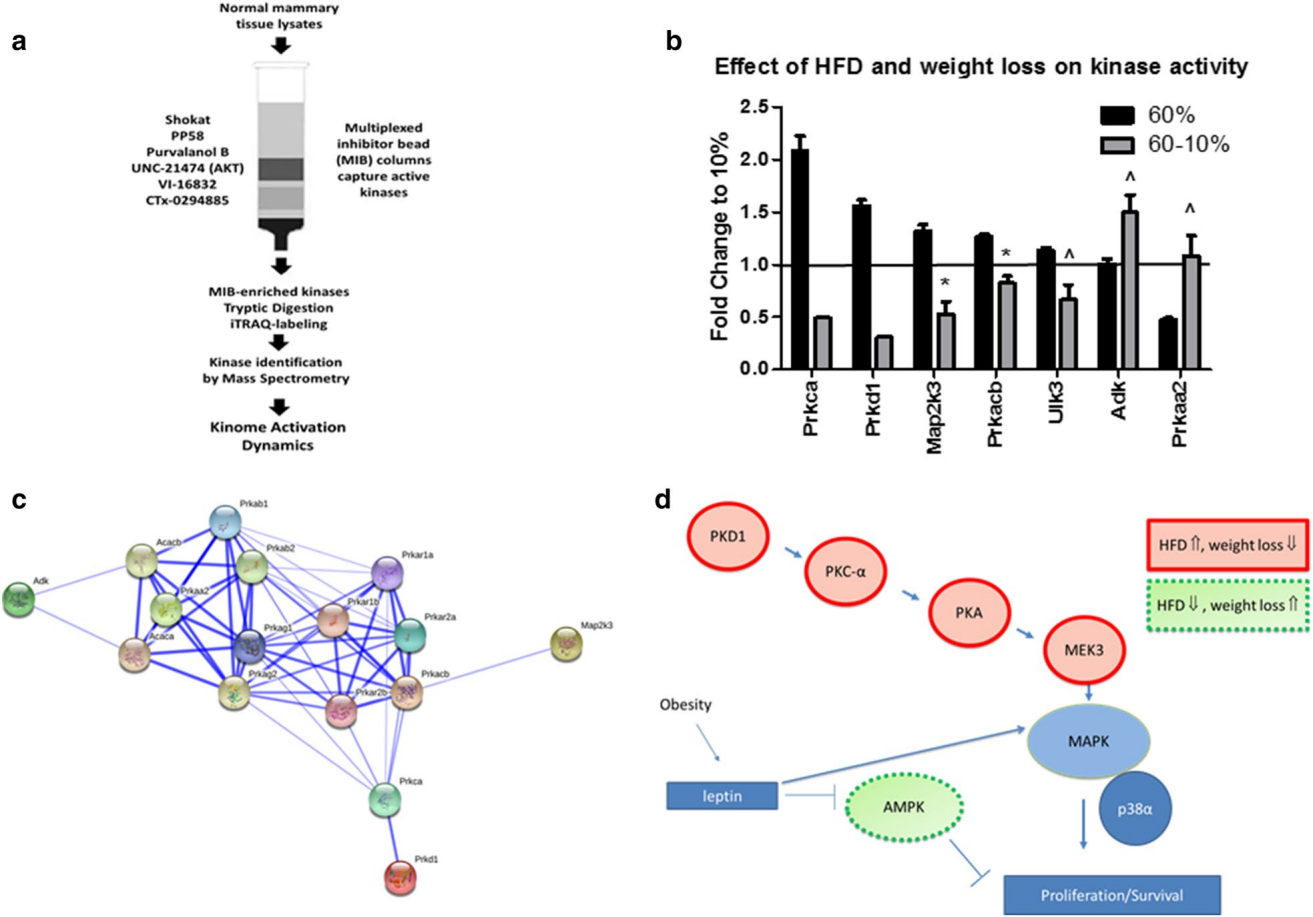
Kinome profiling of unaffected mammary glands revealed regulation of PKD1-PKC-α-PKA-MEK3 and AMPK by diet exposure. To profile the activated kinome, unaffected mammary glands from mice at 15 weeks of age were collected for multiplexed inhibitor bead (MIB) affinity chromatography and mass spectrometry analysis. **a** MIBs consist of mixtures of Sepharose beads with covalently immobilized, linker-adapted, broad pan-kinase inhibitors (listed on *left* of column) are designed to capture kinases in the active state in a reproducible and reliable assay. Two to four samples were pooled into a total of 3 runs per diet group. (N = 8 mice in each diet group). All kinase activity is normalized to 10 %-fed controls. **b** Mean kinase activity is presented to compare kinases in the unaffected mammary glands that were significantly different between mice in the 60 % group and 60–10 % group. *P < 0.005, ^P < 0.05, 60 % vs. 60–10 %) In **b**, no *error bar* is present in pooled samples when kinases were down-regulated below level of detection and only 1 run detected activity. **c**, **d** Protein–protein interactions of significantly altered kinases in unaffected mammary gland of mice on 60–10 % diet compared to mice on 60 % diet. **c** Search Tool for the Retrieval of Interacting Genes/Proteins (STRING version 10) was used to visualize known protein–protein interactions between significantly regulated kinases. Confidence view was shown. Stronger associations are represented by thicker lines. **d** Cartoon of a subset of kinases regulated by HFD and reversed by weight loss and the contribution of obesity-induced leptin signaling

MIB/MS kinome analysis revealed dramatic regulation of kinases by HFD and diet-induced weight loss. Global analysis of kinase activity in unaffected mammary glands was carried out using multiplexed inhibitor beads (MIBs), which consist of mixtures of Sepharose beads covalently bound to various linker adapted, type I pan-kinase inhibitors. MIBs have been shown to preferentially capture kinases in their active conformation to reproducibly measure global dynamic changes in kinase activity [34–36] (Fig. 5a). MIBs have been previously validated using Western immunoblot and kinase assays in breast cancer cell lines and patient samples [34]. Using MIBs and subsequent mass spectrometry (MIB/MS) analysis, we identified a total of 155 kinases from unaffected mammary glands of C3(1)-Tag mice at 15 weeks of age (Additional file 3). Individual runs of 2–4 pooled samples in each run are displayed by kinase family for 60 %-fed mice (Additional file 3a) and 60–10 %-fed mice (Additional file 3b), both groups were normalized to 10 %-fed controls. Means of the runs for the 60 and 60–10 %-fed mice are displayed (Additional file 3c). Additional file 4a shows a kinase signature defining a reprogrammed kinome in response to HFD-induced changes to the mammary gland microenvironment prior to average tumor latency. Six kinases displayed increased activity by >1.5 fold when normalized to control mice on 10 % diet, while the activity of four kinases were decreased to <0.5 fold of kinases activity detected in mammary glands from mice fed 10 % diet (Additional file 4a). The six kinases elevated by HFD included protein kinase C alpha type (KPCA, *Prkca*), bone morphogenetic protein-2-inducible protein kinase (BMP2 K, *Bmp2* *K*), phosphatidylinositol 3-kinase catalytic subunit type 3 (PK3C3, *Pik3c3*), serine/threonine-protein kinase D1 (KPCD1, *Prkd1*), serine/threonine-protein kinase MARK1 (MARK1, *mark1*), and mast/stem cell growth factor receptor Kit (KIT, *Kit*). The 4 kinases decreased by HFD included 5′-AMP-activated protein kinase catalytic subunit alpha-2 (AAPK2, *Prkaa2*), interleukin-1 receptor-associated kinase 1 (IRAK1, *Irak1*), fructosamine-3-kinase (FN3 K, *Fn3* *k*), and epithelial discoidin domain-containing receptor 1 (DDR1, *Ddr1*) in unaffected mammary glands (Additional file 4a).

Weight loss resulted in decreased expression of all kinases that were elevated in HFD-fed mice, since no kinases in the diet switch group increased more than 1.5 fold when normalized to mice on 10 % diet (Additional file 3b). Fourteen kinases from the diet switch group showed more than a 1.25-fold increase in activity when normalized to mice on 10 % diet. In the diet-switch group, five kinases decreased to more than 0.5 fold of the 10 % diet mice (Additional file 4b).

When directly comparing activity of kinases from unaffected mammary glands isolated from mice on HFD versus diet switch groups, several important kinases were discovered to be regulated by HFD and inversely regulated by weight loss (Fig. 5b). Five kinases that were elevated by HFD feeding and decreased by weight loss included KPCA (*Prkca*), serine/threonine-protein kinase D1 KPCD1 (*Prkd1*), dual specificity mitogen-activated protein kinase kinase 3 (MP2K3) MEK3 (*Map2k3,* P = 0.0044), PKA (*Prkacb,* P = 0.0035), and unc-51 like kinase 3 ULK3 (*Ulk3,* P = 0.034). In contrast, two kinases were unchanged or reduced by weight gain and significantly increased by weight loss, respectively, including adenosine kinase ADK (*Adk,* P = 0.045) and 5′-AMP-activated protein kinase catalytic subunit alpha-2 (AAPK2/AMPK (*Prkaa2,* P = 0.037).

## Discussion

One-third of US population is obese and another third is overweight [37]. Considering the high prevalence, obesity could be a target for breast cancer prevention with effective intervention strategies including weight loss, dietary modification, and/or pharmacological approaches. Epidemiologic observations have demonstrated increased BBC risk in premenopausal women with high BMI [16, 38]. BBC is also detected at a high prevalence in African Americans, a group more susceptible to both obesity and weight retention after pregnancy, a period most likely in early adulthood [16]. Indeed, weight loss induced by decreased dietary fat intake in early-stage breast cancer patients has been shown to improve the rate of relapse-free survival [39]. In murine studies, groups including Cleary et al. and Hursting et al. have demonstrated that weight loss induced through caloric restriction protected against the development of mammary tumors [23, 40–42]. We previously reported that HFD-induced tumor progression was reversed by weight loss in a life-long diet exposure study [20]. However, no group has focused on the adult window of susceptibility, a time when most Americans gain weight [37]. Thus, BBC in humans and varied murine models is responsive to energetics and body composition status, but the mechanisms remain unclear.

Herein, we aimed to focus solely on the early adult window of susceptibility and modification of risk factors that could contribute to tumor progression. C3(1)-Tag mice were fed LFD or HFD diets starting at 8 weeks of age, which is after pubertal development and is considered early adulthood in the murine life cycle. Using this unique BBC GEMM, we demonstrated that HFD-feeding in adulthood drove aggressive tumor formation, which resulted in a significant reduction in tumor latency. Importantly, weight loss prior to average tumor onset significantly delayed tumor latency, reversing the effects of HFD. There were no effects on tumor initiation in C3(1)-Tag mice as there were no diet-induced differences in initiating cells expressing Tag. Therefore, data suggest that HFD doesn’t play a dramatic role in the very early regulation of tumor initiating cells in C3(1)-Tag mice, but rather the progression through hyperplasia to DCIS to detectable malignancy.

The normal microenvironment can suppress tumor progression, but when the homeostasis is interrupted (e.g. by obesity, etc.), the microenvironment can promote tumor growth [24]. Therefore, histopathologic analysis was undertaken on unaffected mammary glands isolated from mice at 15 weeks of age before frank tumors could be detected by palpation. Analysis revealed significantly increased ADH and DCIS after just 7 weeks of HFD exposure. Mice that lost weight and adiposity secondary to diet switch were significantly protected from HFD-induced ADH and DCIS development. The number of invasive carcinomas was also decreased by weight loss compared to HFD-fed, although this reduction was not statistically significant. The fact that ADH and DCIS were increased by HFD and reversed to the level of lean mice by weight loss indicated that adiposity could affect already initiated proliferating cells during this unique window of susceptibility (e.g. from initiated cancer stem cells to ADH, or from ADH to DCIS, and from DCIS to IDC) [33]. Thus, the pathological changes detected in the mammary gland at this critical period of tumorigenesis indicated that the progression of basal-like tumors can be delayed or reversed by HFD and weight loss.

Leptin is a hormone produced by adipocytes in proportion to adiposity. A meta-analysis reported that women with breast cancer have elevated plasma leptin levels [43]. High leptin receptor mRNA expression in breast cancer tissue was also established to predict poor prognosis in patients with high serum leptin levels [44]. Leptin may drive BBC through maintaining cancer stem-like properties in orthotopically transplanted mice [45]. Leptin signaling also induced breast tumor cell proliferation in both human and mouse cell lines [46, 47]. Considering the crucial role of leptin signaling in breast cancer in both mouse and human reports, weight loss-associated changes in leptin concentrations could play an important role in BBC prevention. Weight gain in early adulthood increased circulating leptin concentrations and weight loss reversed the leptin levels to those detected in LFD-fed controls. Other contributors often associated with obesity-induced carcinogenesis, including glucose and insulin, were not altered by diet exposures.

Nearly half of the all molecularly targeted cancer therapeutics is kinase inhibitors [48]. Using novel kinome profiling, our results from unaffected mammary glands of mice captured 155 activated kinases from all major kinome subfamilies. HFD feeding and weight loss up- or down-regulated several kinases that interacted with each other (Fig. 5c). Our analysis focused on 7 kinases specifically *reciprocally* regulated by HFD and diet-switch. The kinase that was most elevated by HFD feeding and displayed the greatest decrease in activity after weight loss was a serine/threonine protein kinase (KPCA, *Prkca*), known as PKC-α. Importantly, PKC-α is a marker for breast cancer aggressiveness [49]. Recent studies have identified PKC-α as over-expressed in triple negative breast cancer cells expressing stem-like properties [50]. Inhibitors targeting PKC-α and cMet, another kinase that we reported to be regulated by HFD-induced exposure and weight loss [20, 21], decreased triple-negative breast tumor growth in murine models [50].

An important upstream regulator of PKC-α was demonstrated to be the second most potently upregulated kinase by HFD and dramatically reduced by weight loss, the serine/threonine-protein kinase D1 (KPCD1, *Prkd1)*, also known as PKD1. PKD1 has been shown to increase cell proliferation in breast, prostate, salivary tumors and pancreatic cancers [51, 52]. PKD1 also reduced serum- and anchorage-dependence for proliferation and survival in vitro and drove tumorigenesis in xenograft models of mammary tumors [53]. Borges et al. demonstrated that PKD1 is expressed in cells of the unaffected mammary gland, and is necessary for preventing epithelial-to-mesenchymal transition and invasive carcinoma [54]. Of great relevance to studies presented herein, PKD1 is one of the *few* genes identified as regulator of obesity in human populations. Through genome-wide association studies (GWAS), PKD1 was identified as a loci associated with human obesity, especially in obesity prevalent young adults [55], which is similar to the window of diet exposure and latency in this study. Clearly, PKD1 must be further investigated, especially its role in obesity-associated breast tumorigenesis. Downstream of PKD1 and PKC-α is cAMP-dependent protein kinase catalytic subunit beta (KAPCB, Prkacb), also known as PKA. PKA activity was also increased by HFD and decreased by weight loss. Elevated PKA activity in the mammary epithelium generated tumors in a murine model and was associated with BBC and poorer outcomes in patients [56].

PKD1, PKC-α and PKA all act through the MAPK kinase family. The MAP kinase kinase family member the dual specificity mitogen-activated protein kinase kinase 3 (MP2K3, *Map2k3*), also known as MEK3 or MKK3, was also significantly upregulated. MEK3 is increased by mutant p53 [57], and led to cell proliferation and survival through increased oncogene RAS expression, as well as activation of p38α (MAPK14) [58]. Inhibition of MEK3 led to reduction in cell proliferation and apoptosis [59], and knockdown of MEK3 led to reduced cell viability as well as increased susceptibility to chemotherapeutic agents in vitro and in vivo [59]. Interestingly, MEK3 is also linked to obesity [60] and lipotoxicity [61] in human populations, as well as diabetes in a murine model [62]. In sum, Fig. 5d demonstrates how weight loss reversed the HFD-induced activation of PKC-α, PKD1, PKA, and MEK3 in unaffected mammary glands, which may lead to inactivation of MAPK/p38α pathway, resulting in delayed tumor latency in mice lost weight compared to mice fed HFD.

A final kinase moderately but significantly up-regulated by HFD and down-regulated by weight loss was the serine/threonine protein kinase unc-51 like kinase 3, ULK3 (*Ulk3*). ULK3 regulates the developmental and oncogenic pathway of sonic hedgehog (SHH) signaling and autophagy. ULK3 is over-expressed in certain cancer cell lines [63]. Since it is significantly reduced with weight loss after weight gain, ULK3 may be a promising target.

Two kinases were regulated in the inverse direction: these were reduced by HFD and increased with weight loss after diet switch. A kinase whose activity was reduced with HFD to less than 40 % of lean mice on LFD, and then reversed to control levels with weight loss was the alpha 2 catalytic subunit of AMP-activated protein kinase (AAPK2, *Prkaa2*) (Fig. 5d). AMPK is an energy-sensing kinase that controls nutrient metabolism [64]. Leptin is known to inhibit AMPK’s action [65]. Consistent with increased activated AMPK by weight loss in our results, activation of AMPK has been suggested to be a target for cancer prevention and treatment [66]. A low incidence of cancers in diabetic patients on metformin is likely due to the drug’s anti-proliferative effect through activation of AMPK [67]. Breast cancer patients on metformin have been found to have a lower proportion of higher stage tumors than control patients [68]. Importantly, metformin can inhibit cell growth in basal-like cancer cells [69]. AMPK merits further study in BBC cancer prevention.

A limitation in the interpretation of our studies is that we induced weight loss after HFD exposure using a common protocol of diet switch. While epidemiologic and experimental evidence overwhelmingly support a strong role for obesity in breast cancer risk, some reports suggest that HFD exposure alone (in the absence of obesity) is enough to alter the mammary microenvironment and increase breast cancer risk [22, 70–72]. The most common HFD used in research is lard-based, which contains high levels of saturated fatty acids, known activators of the toll like receptor 4 (TLR4) pathway [73–76], a contributor to breast cancer [22]. Thus, in our study design it is unknown if diet exposure itself or obesity-associated alterations contribute to BBC progression. Future studies include modification of diet including caloric restriction to determine the contribution of diet alone versus changes in adiposity and leptin as reported herein.

## Conclusions

One-third of US adults are obese and two-thirds are overweight, underscoring a critical need to reduce breast cancer risk, especially triple negative breast cancers (TNBC) that are significantly associated with obesity. Obesity was recently recognized by the American Medical Association as a disease [77] and the American Society of Clinical Oncology just published a position statement on their recognition of and commitment to reducing obesity-associated cancer [78]. Lifestyle components, like obesity, play an important role in cancer initiation and progression [24]. While biological models of cancer have traditionally emphasized cell-autonomous characteristics, it is clear that changes in the microenvironment are also necessary [79]. Herein we have identified the adipokine leptin and relevant kinases that correlate with tumor latency and pre-neoplastic lesion formation in the mammary gland. Future studies to further elucidate the molecular links between cells of the microenvironment and unique obesity-regulated factors will be transformative, with significant potential to influence obesity prevention or dietary recommendation initiatives and may yield biomarkers of risk for future study. Improved understanding of biological mechanisms could also yield prevention strategies to address racial disparities since BBC is highly prevalent in young, African American and Hispanic women, contributing to disparities in cancer mortality [19].

## Methods

Reagents. Rat/Mouse Insulin ELISA kit was obtained from Millipore (EZRMI-13 K; EMD Millipore, Billerica, MA, USA). Anti-SV40-Tag (sc-20800) was obtained from Santa Cruz (Santa Cruz, Santa Cruz, CA). Biotin-SP (longer spacer) AffiniPure Goat anti-Rat IgG was from Jackson ImmunoResearch (#112-065-167, Jackson ImmunoResearch Inc. West Grove, PA, USA).

### Animals and diet

Animal studies were performed with approval and in accordance with the guidelines of the Institutional Animal Care and Use Committee at the University of North Carolina at Chapel Hill. Animals were cared for according to the recommendations of the Panel on Euthanasia of the American Veterinary Medical Association. The veterinary care provided at UNC is in compliance with the Public Health Service Policy on Humane Care and Use of Laboratory Animals and meets the National Institutes of Health standards as set forth in the Guide for the Care and Use of Laboratory Animals (DHHS Publication No. (NIH) 85-23 Revised 1985). The animal facility is Association for Assessment and Accreditation of Laboratory Animal Care (AAALAC) approved and is responsible for the health and husbandry of animals. UNC also accepts as mandatory the PHS Policy on Humane Care and Use of Laboratory Animals be Awardee Institutions and NIH Principles for the Utilization and Care of Vertebrate Animals Used in Testing, Research, and Training. During breeding and after weaning at 3 weeks old, female C3(1)-Tag mice were put on Prolab Isopro RMH 3000 from LabDiet (St. Louis, MO, USA) until they were 8 weeks old when they were started on defined diets. Matched defined diets from Research Diets Inc. (New Brunswick, NJ, USA) provided 10 % kcal (“10 %”, LFD) and 60 % kcal (“60 %”, HFD) derived from fat. Details of diet in Sundaram et al. [21]. At 8 weeks of age, mice were randomly assigned to LFD 10 % (N = 28) and HFD 60 % (N = 59) diet groups. After 3 weeks on diet, at 11 weeks of age, N = 28 mice on 60 % were switched to 10 % diet (Study 1, Fig. 1a). A second set of mice were similarly initiated on 10 % (N = 8) and 60 % (N = 20) at 8 weeks of age. At 11 weeks of age, N = 11 of 60 %-fed were switched to 10 % as above. This second cohort was sacrificed at 15 weeks of age prior to average tumor latency and unaffected mammary glands were isolated (Study 2, Fig. 3a).

### Tumor latency, number, and progression

Tumor latency was defined as age at detection of first tumor in weeks. After detection of the first tumor, tumor volumes were monitored weekly over 3 weeks using calipers to measure the width (short diameter) and length (long diameter) in millimeter for each tumor. The tumor volumes were calculated using the formula: length × width^2^ × 0.5. The total number of tumors per mouse was counted at sacrifice for total tumor burden.

### Body weight and composition

Body weight was measured prior to starting mice on diet and weekly until sacrifice. Body composition including lean mass, fat mass, free water content, and total water content was also measured at 8 (diet start), 11 (diet switch), 13, and 15 weeks of age, as well as at sacrifice using quantitative magnetic resonance whole body composition analyzer (Echo Medical Systems, Houston, TX, USA). Fat mass is presented as percent fat mass over total body weight [20, 21].

### Metabolic parameters and plasma

Blood glucose was measured on mice fasted for 6 h prior to start of diet, at diet switch (3 weeks on diet), 2 weeks after diet switch, and at sacrifice using a Bayer contour blood glucose monitor (Bayer HealthCare LLC, Tarrytown, NY, USA). Plasma was collected at different time points. Plasma leptin was measured using the MILLIPLEX MAP mouse angiogenesis/growth factor magnetic bead panel—cancer multiplex assay (EMD Millipore, Billerica, MA, USA). Plasma insulin concentrations were measured using Rat/Mouse Insulin ELISA kit (EZRMI-13 K; EMD Millipore, Billerica, MA, USA). The homeostasis model assessment was used to calculate the approximate insulin resistance (HOMA_IR_) using the formula (blood glucose (mg/dl at sacrifice) × plasma insulin concentration (ng/ml)/405) [21, 80].

### Histological staining

Portions of the unaffected fourth mammary glands isolated from mice at 15 weeks of age in study 2 (Fig. 3a) were formalin-fixed and paraffin-embedded for immunohistochemistry (IHC) and H&E staining. Anti-SV40-Tag (1:250) IHC staining was conducted and analyzed as previously described [21, 31, 76]. Briefly slides were scanned into the Aperio Scanscope CS system (Aperio Technologies, Vista, CA, USA) at a magnification of 40× and quantified using the Aperio Imagescope software. The slides were analyzed using the algorithms as described [31]. Random areas from sections (N = 6) were quantified and averaged per animal. Microenvironment pathological analysis of H&E staining was completed by a certified veterinary pathologist (S.M.). Sections of unaffected mammary from study 2 were analyzed for the presence of normal ducts, ADH, DCIS, and IDC.

### MIB/MS kinome analysis

Multiplexed inhibitor bead (MIB) affinity chromatography was completed to measure activated kinases as described previously [36]. Briefly, unaffected mammary glands from study 2 were pulverized and lysed in MIB lysis buffer, and 1 % of phosphatase inhibitor cocktail 2 and 3 (Sigma-Aldrich)] [34]. Two to four samples were pooled together for a total protein content of 2.5 mg. Tissue lysates were passed through a column of layered inhibitor-conjugated beads consisting of sepharose-conjugated Shokat, AGCbead (UNC-21474), Purvalanol B, PP58, VI-16832 and CTx-0294885 (Fig. 5a) [34]. Kinase-bound inhibitor beads were washed eluted, reduced, alkylated, and concentrated before chloroform/methanol extraction. Protein pellets were resuspended in 50 mM HEPES (pH 8.0), digested, with trypsin (Promega), labeled with iTRAQ reagent (AB SCIEX, Framingham, MA) and cleaned with PepClean C18 spin columns (Thermo Scientific).The details for mass spectrometry (MS) have been prescribed previously [81]. Thermo Orbitrap Elite mass spectrometer with a nanoAquity UPLC system (Waters Corp.) was used for data acquisition. Briefly, peptides were first trapped in a 2 cm trapping column and separated on a 20 cm column at room temperature. Samples were run in a full 200 min gradient. Spectra were searched against the Uniprot/Swiss-Prot database with Sequest HT on Proteome Discoverer software 1.4. Only peptides with high confidence were considered for quantitation.

### Statistical analysis

Data are expressed as mean and standard error of the mean (SEM). Percentage of tumor-free mice amongst the diet groups was compared with Kaplan–Meier analyses. Log rank and Chi square test were used to investigate differences among groups for tumor latency. Continuous variables for two groups were compared using Student’s t-tests. Continuous variables for more than two groups were compared using one way analysis of variance (ANOVA) with Tukey’s post hoc test. The correlation of latency with leptin level at different time points was computed via Spearman Rank correlation. Analyses were performed using SAS Version 9.3 (SAS Institute, Cary NC USA) or GraphPad Prism 5 software (GraphPad Software, Inc. La Jolla, CA, USA). P values <0.05 were considered statistically significant.

## Additional files

10.1186/s12935-016-0300-y Tumor burden and growth were not affected by diet. a. Tumor burden was quantified at sacrifice. b. Tumor volume was measured by calipers at detection and sacrifice. (N = 28 10 %; N = 31 60 %; N = 29, 60–10 %).
10.1186/s12935-016-0300-y Measures of glucose intolerance were not altered by diet. A and b. Fasting and plasma insulin concentrations were measured in 6 h fasted mice at time points indicated. c. Homeostasis model assessment of insulin resistance (HOMAIR) was calculated. (N = 12 10 %, N = 12 60 %; N = 13 60–10 %).
10.1186/s12935-016-0300-y Kinome profiling revealed significant regulation of pathways by HFD that were reversed with weight loss. A and b. Quantitative comparison of kinases in unaffected mammary tissues from mice using MIB/MS was conducted. Legend indicates three iTRAQ runs with 2–4 samples pooled per group per run. The graphs indicates quantitative changes in kinase activity as a ratio of mice fed 60 % (a) or 60–10 % (b) diet relative to mice fed 10 % diet group. Ratio <1 denotes decreased kinase activity and >1 increased kinase activity. Kinase families are indicated (AGC: Containing PKA, PKG, PKC families; CAMK: Calcium/calmodulin-dependent protein kinase; CK1: Casein kinase 1; CMGC: Containing CDK, MAPK, GSK3, CLK families; STE: Homologs of yeast Sterile 7, Sterile 11, Sterile 20 kinases; TK: Tyrosine kinase; TKL: Tyrosine kinase-like). c. Mean kinase activity is reported for mice fed 60 % diet (dark grey) or 60–10 % diet (light grey) compared to mice on 10 % diet group. Error bars are not indicated for clarity. Statistically significant comparisons are reported in Fig. 5d.
10.1186/s12935-016-0300-y Kinome analysis revealed dramatic regulation of kinases by HFD and diet-induced weight loss. a. HFD (60 %-fed) mammary gland kinase activity of greater than 1.5 or less than 0.5 fold compared to 10 %-fed is presented. b. Weight loss (60–10 %-fed) mammary gland kinase activity of greater than 1.25 or less than 0.5 fold compared to 10 %-fed is presented. In b, no error bar is present in pooled samples when kinases were down-regulated below level of detection and only 1 run detected activity.

## Abbreviations

ADH: atypical ductal hyperplasia
AMPKa: 5′-AMP-activated protein kinase catalytic subunit alpha
ANOVA: analysis of variance
BBC: basal-like breast cancer
DCIS: ductal carcinoma in situ
GEMM: genetically engineered mouse model
GWAS: genome-wide association studies
HFD: high fat diet
IDC: invasive ductal carcinoma
IHC: immunohistochemistry
LFD: low fat diet
MAPK: mitogen-activated protein kinase
MEK3: dual specificity mitogen-activated protein kinase kinase 3
MIB: multiplexed inhibitor bead
MS: mass spectrometry
PKA: cAMP-dependent protein kinase catalytic subunit alpha
PKC-α: protein kinase C alpha type
PKD1: serine/threonine-protein kinase D1
SHH: sonic hedgehog
ULK3: unc-51 like kinase 3
