# Supplementary figures and images for: Weight loss reduces basal-like breast cancer through kinome reprogramming
Source: Cancer Cell Int. 2016 Apr 1;16:26. doi: 10.1186/s12935-016-0300-y (PMC4818517; doi:10.1186/s12935-016-0300-y)

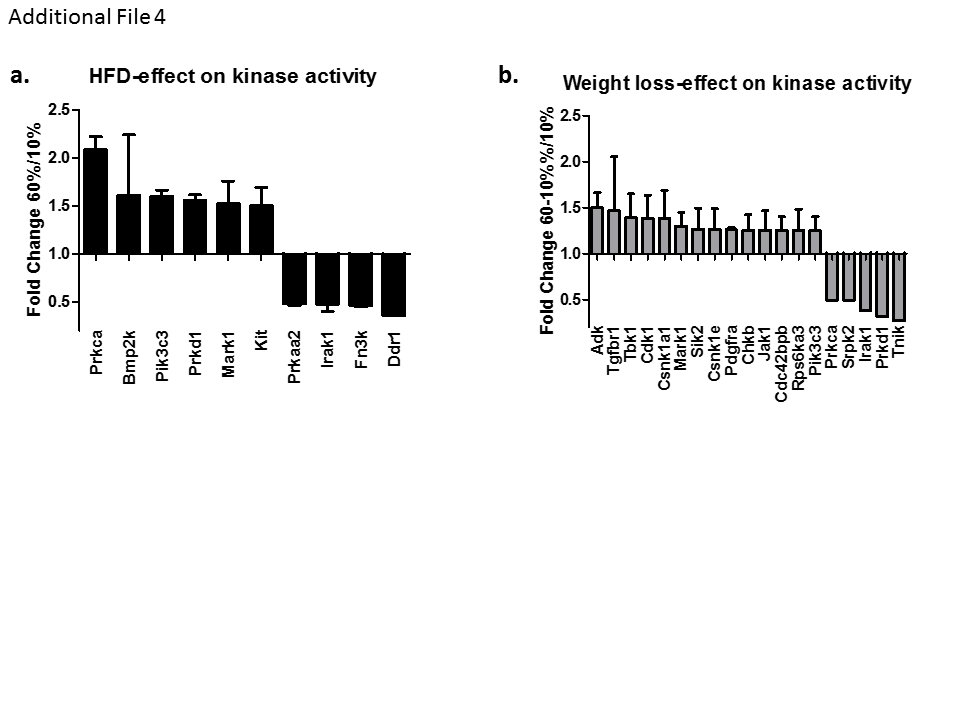

Supplement: Supplementary file 1 — 10.1186/s12935-016-0300-y Tumor burden and growth were not affected by diet. a. Tumor burden was quantified at sacrifice. b. Tumor volume was measured by calipers at detection and sacrifice. (N = 28 10 %; N = 31 60 %; N = 29, 60–10 %). [file 12935_2016_300_MOESM1_ESM.tif]

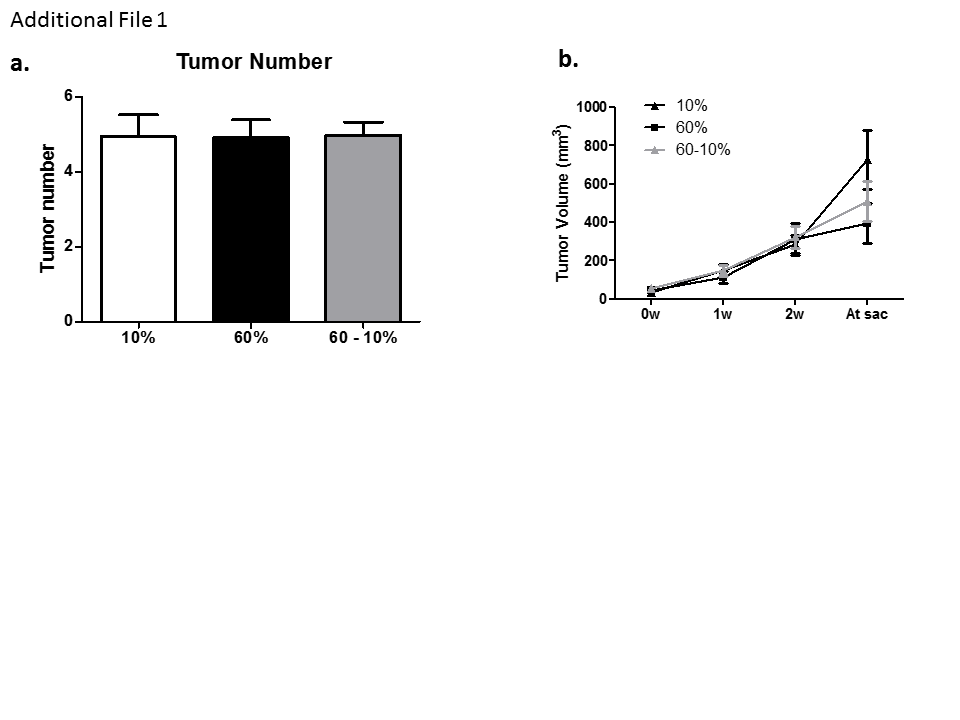

Supplement: Supplementary file 2 — 10.1186/s12935-016-0300-y Measures of glucose intolerance were not altered by diet. A and b. Fasting and plasma insulin concentrations were measured in 6 h fasted mice at time points indicated. c. Homeostasis model assessment of insulin resistance (HOMAIR) was calculated. (N = 12 10 %, N = 12 60 %; N = 13 60–10 %). [file 12935_2016_300_MOESM2_ESM.tif]

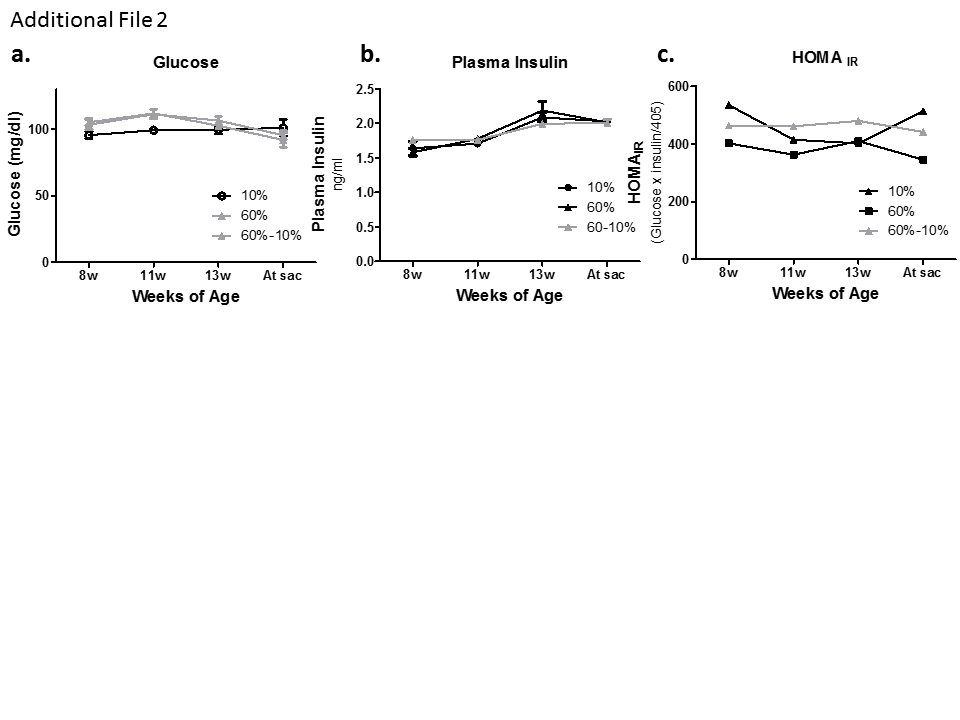

Supplement: Supplementary file 3 — 10.1186/s12935-016-0300-y Kinome profiling revealed significant regulation of pathways by HFD that were reversed with weight loss. A and b. Quantitative comparison of kinases in unaffected mammary tissues from mice using MIB/MS was conducted. Legend indicates three iTRAQ runs with 2–4 samples pooled per group per run. The graphs indicates quantitative changes in kinase activity as a ratio of mice fed 60 % (a) or 60–10 % (b) diet relative to mice fed 10 % diet group. Ratio <1 denotes decreased kinase activity and >1 increased kinase activity. Kinase families are indicated (AGC: Containing PKA, PKG, PKC families; CAMK: Calcium/calmodulin-dependent protein kinase; CK1: Casein kinase 1; CMGC: Containing CDK, MAPK, GSK3, CLK families; STE: Homologs of yeast Sterile 7, Sterile 11, Sterile 20 kinases; TK: Tyrosine kinase; TKL: Tyrosine kinase-like). c. Mean kinase activity is reported for mice fed 60 % diet (dark grey) or 60–10 % diet (light grey) compared to mice on 10 % diet group. Error bars are not indicated for clarity. Statistically significant comparisons are reported in Fig. 5d. [file 12935_2016_300_MOESM3_ESM.tif]

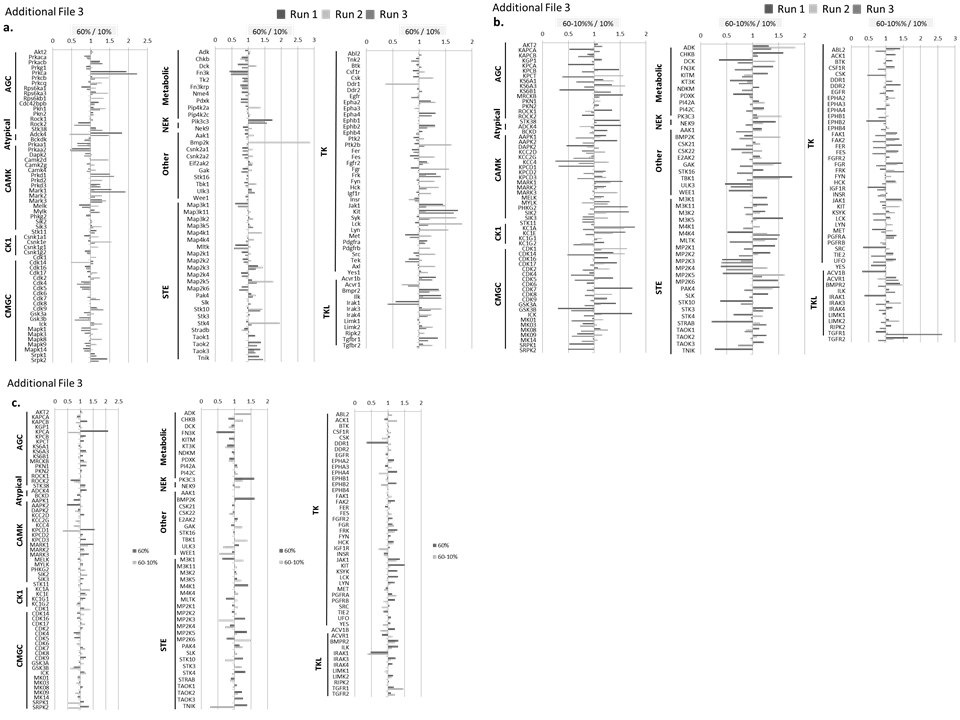

Supplement: Supplementary file 4 — 10.1186/s12935-016-0300-y Kinome analysis revealed dramatic regulation of kinases by HFD and diet-induced weight loss. a. HFD (60 %-fed) mammary gland kinase activity of greater than 1.5 or less than 0.5 fold compared to 10 %-fed is presented. b. Weight loss (60–10 %-fed) mammary gland kinase activity of greater than 1.25 or less than 0.5 fold compared to 10 %-fed is presented. In b, no error bar is present in pooled samples when kinases were down-regulated below level of detection and only 1 run detected activity. [file 12935_2016_300_MOESM4_ESM.tif]
